# Controlling Osteogenic Stem Cell Differentiation via Soft Bioinspired Hydrogels

**DOI:** 10.1371/journal.pone.0098640

**Published:** 2014-06-17

**Authors:** Amit K. Jha, Wesley M. Jackson, Kevin E. Healy

**Affiliations:** 1 Department of Bioengineering, University of California, Berkeley, California, United States of America; 2 Department of Materials Science and Engineering, University of California, Berkeley, California, United States of America; University of California at Davis, United States of America

## Abstract

Osteogenic differentiation of human mesenchymal stem cells (hMSCs) is guided by various physical and biochemical factors. Among these factors, modulus (i.e., rigidiy) of the ECM has gained significant attention as a physical osteoinductive signal that can contribute to endochondral ossification of a cartilaginous skeletal template. However, MSCs also participate in intramembranous bone formation, which occurs *de novo* from within or on a more compliant tissue environment. To further understand the role of the matrix interactions in this process, we evaluated osteogenic differentiation of hMSCs cultured on low moduli (102, 390 or 970 Pa) poly(N-isopropylacrylamide) (p(NIPAAm)) based semi-interpenetrating networks (sIPN) modified with the integrin engaging peptide bsp-RGD(15) (0, 105 or 210 µM). Cell adhesion, proliferation, and osteogenic differentiation of hMSCs, as measured by alkaline phosphatase (ALP), runt-related transcription factor 2 (RUNX2), bone sialoprotein-2 (iBSP), and osteocalcien (OCN) protein expression, was highest on substrates with the highest modulus and peptide concentrations. However, within this range of substrate stiffness, many osteogenic cellular functions were enhanced by increasing either the modulus or the peptide density. These findings suggest that within a compliant and low modulus substrate, a high affinity adhesive ligand serves as a substitute for a rigid matrix to foster osteogenic differentiation.

## Introduction

The field of mechanobiology has recently focused on how the substrate modulus affects the differentiation of various stem cell populations, including human mesenchymal stem cells (**hMSCs**). The seminal work by Engler *et al.* [1] demonstrated that cell fate can be manipulated by altering the modulus (i.e., rigidity) of the substrate and reported that osteogenic differentiation was maximized on rigid matrices (25–40 kPa). More recent studies on osteogenic differentiation have confirmed this observation on materials with moduli ranging from 5 kPa to 40 kPa [2], [3], [4], [5]. The findings from these studies have drawn substantial attention to the role of matrix physical parameters as mediators of stem cell behavior to guide tissue development. Likewise, appropriate engineering of these matrix parameters has been essential for developing strategies to improve the success of tissue-engineered biomaterial-based cell transplantation therapies [6].

It is noteworthy that bone development *in vivo* occurs via two distinctly different process: endochondral and intramembranous ossification [7]. During endochondral ossification, terminal osteoblast differentiation occurs in a cartilagenous template, which has a high modulus relative to the surrounding soft tissues, and it is gradually replaced by bone as the tissue is remodeled and mineralized [8]. By contrast, intramembranous ossification occurs during development by direct differentiation of MSCs into bone forming osteoblasts within a soft mesenchymal matrix [7], where the shear modulus of embryonic tissues has been estimated to be in the range of 100–1000 Pa [9], [10]. Previous studies have also demonstrated that *in vitro* osteogenic differentiation can occur on compliant materials [11], [12], and *in vivo* osteogenic differentiation has also been observed in soft substrates over the range of 50 to 500 Pa [13], [14], which is similar to the stiffnesses of matrices in the developing embryo where intramembranous ossification occurs. Thus, these studies indicate that other properties of the ECM, such as the presentation and density of adhesion ligands and biochemical factors, are sufficient to drive osteogenic differentiation of hMSCs *in vivo* in the absence of a rigid matrix or tissue substrate [15], [16]. However, it is unknown how these additional material properties of the matrix may compensate for “non-endochondral” substrate stiffness to encourage osteogenic differentiation of MSCs within a compliant matrix or tissue substrate. Binding between adhesion domains within the matrix and integrins at the cell surface initiates intracellular signaling pathways that transmit mechanical feedback from the underlying matrix to modulate hMSC differentiation [17]. Thus, the magnitude of mechanical feedback depends on the density of integrin binding peptides, integrin receptor type and number, matrix stiffness, and osteoinductive factors, all of which are sufficient to modulate osteogenesis in hMSCs [11].

In this study we aimed to test the hypothesis that osteogenic differentiation of hMSCs on matrices with low moduli (e.g. stiffnesses ranging 100–1000 Pa) can be enhanced by increasing the adhesive peptide density of the substrate. For this study, it was a prerequisite to create materials with defined properties to study the interplay between internal and external determinants of stem cell fate. Accordingly, we have assessed the osteogenic differentiation function of hMSCs cultured on a poly(N-isopropylacrylamide) (**p(NIPAAm)**) hydrogel system, which has been used previously for independent control of matrix stiffness and adhesive ligand density [13], [18], [19], [20], [21] and has also demonstrated the ability to support bone formation *in vivo* [13]. We also used a 15 amino acid adhesion ligand containing an Arg-Gly-Asp site that was originally derived from bone sialoprotein (BSP) (CGGNGEP**RGD**TYRAY), which promotes greater cell adhesion compared to shorter RGD- peptides [22], [23], [24], [25], [26], [27] lacking the adjacent residues. This sequence (**bsp-RGD(15)**) been shown to support cell adhesion, spreading, osteogenic differentiation and matrix mineralization [13], [20], [22], [28], [29], [30], [31]. Additionally, a short matrix metalloproteinase-13 (MMP-13) cleavable peptide sequence was used as a crosslinker to tune the mechanical properties and degradation kinetics of the hydrogel. We chose a MMP-13 degradable crosslinker, since this MMP was upregulated in an *in vivo* bone regeneration injury model [13], [14], [32].

## Materials and Methods

### 1. Materials

Polyacrylic acid [450 kDa; p(AAc)], N-isopropylacrylamide (NIPAAm), acrylic acid (AAc), N,N,N,N-tetramethylenediamine (TEMED; Chemzymes Ultrapure grade) were purchased from Polysciences (Warrington, PA). 1-Ethyl-3-(3-dimethylaminopropyl) carbodiimide hydrochloride (EDC), N-hydroxysulfosuccinimide (Sulfo-NHS), acryloyl chloride and N-(e-maleimidocaproic acid) hydrazide (EMCH) were purchased from Sigma Aldrich (Milwaukee, WI). The peptide crosslinker (QPQGLAK) containing an amidated lysine at the C-terminal and the bone sialoprotein-derived, RGD-containing 15 amino acid peptide (CGGNGEPRGDTYRAY; referred to as bsp-RGD(15)) [21], [22], [28], [29], [33], was synthesized by American Peptide (Sunnyvale, CA). Acetone, isopropanol, hydrochloric acid, methyl red, sodium hydroxide, perchloric acid, ammonium hydroxide, sodium chloride, chloramine-T, p-dimethylaminobenzaldehyde (p-DAB), dimethylacetamide (DMAc), triethylamine (TEA), 3,3′,5,5′-tetramethylbenzidine (TMB) substrate and ethanol were obtained from Thermo Fisher Scientific (Waltham. MA). Dialysis membranes (SpectraPor Biotech CE) were purchased from Spectrum Laboratories (Rancho Dominguez, CA). Centrifugal filter devices with 50 kDa average MW cutoff (MWCO) were obtained from Pall Gelman Laboratory (Ann Arbor, MI). Dulbecco's phosphate buffered saline (PBS), Dulbecco's modified Eagle medium (DMEM; high glucose, with L-glutamine, with pyridoxine hydrochloride, without sodium pyruvate), heat-inactivated fetal bovine serum (FBS), penicillin–streptomycin, ascorbic acid, N-(2-hydroxyethyl)piperazine-N'-(2-ethanesulfonic acid) (HEPES) buffer, and sodium pyruvate were purchased from GIBCO BRL (Grand Island, NY). Paraformaldehyde (16% in water) was obtained from Electron Microscopy Sciences (Hartfield, PA). Propidium iodide and Syto 13 were purchased from Genway Biotech, Inc (San Diego, CA). Tetramethyl rhodamine isothiocyanate (TRITC)-conjugated phalloidin, and DAPI were purchased from Millipore (Billerica, MA). Mouse monoclonal anti-STRO-1 IgG antibody and Alexa Fluor 568-labeled goat anti-mouse IgG antibody were obtained from Invitrogen (Carlsbad, CA). Mouse polyclonal anti-collagen type I IgG antibody was obtained from Abcam (Cambridge, MA). All antibodies were reactive against human antigens and diluted in PBS containing 3% bovine serum albumin (BSA; Jackson Immunoresearch, West Grove, PA).

### 2. P(NIPAAm) based sIPNs

P(NIPAAm) based sIPNs were synthesized using previously reported procedures [13], [34]. Briefly, an MMP-13-cleavable peptide (QPQGLAK) was functionalized with acrylate groups on both ends for use as a crosslinker. sIPNs were synthesized by radical crosslinking of NIPAAm and AAc monomers with defined concentrations of p(AAc), acrylated MMP-13 peptide crosslinker, and p(AAc)-*g*-RGD in the primary crosslinked network.

#### 2.1. QPQGLAK diacrylate synthesis

Acrylate groups were functionalized at amines preset at the both ends of the MMP-13-cleavable peptide (i.e., primary amines at the N-terminal lysine and a secondary amine substituted by the manufacturer for the carboxylic acid at the C-terminal glutamine) by the reaction of acryloyl chlorides via addition-elimination mechanism between nucleophilic amine nitrogen and the carbonyl carbon of acryloyl chloride [34], [35]. Briefly, the peptide (QPQGLAK; 165 mg) was dissolved in a mixture of DMAc/TEA and flushed with nitrogen for 5 min. In another vial, acryloyl chloride (128 µl) was dissolved in 6.5 mL DMAc and was added drop-wise to the peptide solution on ice. After four hours of reaction, TEA and DMAc were removed by rotary evaporation. The collected precipitate was then removed from the evaporation, dissolved in ultrapure water (UPW) and dialyzed (100 Da MWCO) against UPW for 48 h. The purified product was lyophilized and stored at −80°C.

#### 2.2. P(AAc)-g-RGD synthesis

Separately, linear chains of polyacrylic acid (pAAc, 450 kDa) were grafted with bsp-RGD(15) using maleimide-thiol coupling chemistry as described previously [34], [35]. Carboxylic groups on p(AAc) were functionalized with maleimide using carbodiimide chemistry. p(AAc) solution in MES buffer (pH 6.5) was reacted with an EMCH/Sulfo-NHS/EDC mixture. After two hours, the reaction mixture was purified using centrifugal filtration tubes (50 kDa MWCO). Then, bsp-RGD(15) was grafted on p(AAc)-*g*-maleimide in sodium phosphate buffer (pH 6.6) by coupling the terminal cysteine of bsp-RGD(15) with maleimide on p(AAc). The final product p(AAc)-*g*-RGD was purified in the centrifugal filtration tubes and then lyophilized.

#### 2.3. sIPNs polymerization

A solution of NIPAAm (5% w/v), AAc, QPQGLAK diacrylate, and p(AAc)-*g*-RGD in PBS was bubbled with nitrogen for 15 minutes to remove dissolved oxygen. sIPN synthesis by radical crosslinking was initiated by the addition of ammonium persulfate (0.8% w/v) and TEMED (8% w/v) and allowed to react for 24 hours under an inert environment. sIPNs stiffness and peptide density was controlled by varying the concentration of peptide crosslinker and p(AAc)-*g*-RGD, respectively. Unreacted monomers, initiators, and other unbound impurities were removed by washing the hydrogel three times with PBS, and subsequently the hydrogel disks were sterilized by rinsing them three times with ethanol (70%) at 37°C. Prior to seeding cells on the sIPN disks, the hydrogels were washed an additional three times with sterilized PBS at 37°C to remove the ethanol.

### 3. Rheological characterization of sIPNs

Viscoelastic properties of the sIPNs were determined by an oscillatory rheometer (MCR300, Anton Paar, Ashland, VA) with 25-mm parallel plates, gap height of 0.5 mm and 5% strain at 37°C and over a range of frequency ranging from 0.001 to 10 Hz. Drying of the sample was prevented by performing the measurements within a humidity-controlled chamber.

### 4. Cell culture, viability and cell adhesion on sIPNs

Human mesenchymal stem cells (hMSCs; Lonza, Walkersville, MD) were cultured at 37°C and 5% CO_2_ in MSC growth media (MSCGM; Lonza Walkersville, MD). Subsequently, hMSCs (passage 5–6) were seeded on the top of the sIPN disks at 10,000 cells/cm^2^.

#### 4.1 Viability assay

sIPN constructs seeded with hMSCs were incubated in growth media for 24 hours, and then cell viability was assessed by live/dead staining with propidium iodide (1∶2000 in PBS) and calcein (1∶1000 in PBS) for 5 min at room temperature. Images were acquired using a two-photon/confocal microscope (Prairie Technologies, Middleton, WI).

#### 4.2 Cell adhesion assays

Cell adhesion was determined by f-actin staining. hMSCs were cultured on sIPNs for 3 days as described above, washed three times with PBS, and fixed with 4% paraformaldehyde in PBS at room temperature for 30 min. Samples were blocked with bovine serum albumin (BSA; 3 wt% in PBS) at room temperature for 30 min and then stained with Rhodamine-labeled phalloidin (1∶200) in the dark for 2 h at room temperature. Prior to imaging, cell nuclei were stained with DAPI (1∶1000) for 5 min at room temperature. Confocal images were acquired using a two-photon/confocal microscope. Confocal images were analyzed using Image J (NIH) to calculate average cell spread area for each hydrogel condition.

#### 4.3 Cell proliferation assays

Cell proliferation was quantified using the colorimetric Alamar blue assay. hMSCs were cultured on sIPNs as described above for 1, 7, 14 or 21 days, and then incubated with growth medium containing 10% Alamar blue for 12 hours. 200 µL of the medium was used to read absorbance at 570 nm, which was compared against the absorbance of hMSCs cultured under identical media conditions, but on tissue-culture polystyrene (TCPS). Cell attachment to the sIPNs was assessed by the cell number at day 1 divided by the number of cells originally seeded to each substrate.

#### 4.4 Response Surface methodology

Image J was used to calculate average cell spread area for each uniform gel condition, and the number of attached cells was calculated using the Alamar blue assay. Data collected from the cell spreading area and number of adherent cells were transformed into response surfaces based on a quadratic fit for the nine data points with respect to two factors, matrix stiffness and peptide concentration, using JMP statistical software (SAS, North Carolina, USA).

### 5. Cell differentiation

Prior to induction of osteogenic differentiation, hMSCs were seeded on sIPNs at 10,000 cells/cm^2^ and cultured in MSC growth medium for 7 days. Then, the hMSCs were cultured for up to 21 additional days in osteogenic medium consisting of DMEM with β-glycerophosphate (10 mM), ascorbic-2-phosphate (50 µM), dexamethasone (100 nM), and FBS (10%).

#### 5.1. Quantitative analysis of STRO-1 expression

At 0, 7 and 14 days after the start of osteogenic differentiation, the cells were fixed with cold methanol at −20°C and subsequently incubated with anti-STRO-1 mouse monoclonal IgG antibody (1∶50 dilution) at 4°C overnight. For STRO-1 localization, the fixed cells were incubated for 1 hour with Alexa Fluor 568-labeled secondary antibody (1∶100 dilution) at room temperature and then confocal images were taken using a two photon/confocal microscope. For quantification of relative expression of STRO-1, the fixed hMSCs were then incubated with a horseradish peroxidase (HRP) conjugated secondary antibody (1∶100 dilution) for 2 hours at room temperature, washed three times with PBS and incubated with TMB substrate for 5 minutes at room temperature according to the manufacturer's instruction for HRP quantification. After adding the stop solution, colorimetric analysis of the supernatants at 450 nm was performed using an MRX multiplate reader (Dynatech Labs, USA). STRO-1 protein expression was normalized by cell number using the results of the proliferation assay at each time point.

#### 5.2. Assessment of alkaline phosphatase (ALP) activity

ALP Colorimetric Assay Kit (BioVision, Inc., Milpitas, CA) was used to measure ALP activity according to the manufacture's protocol. After washing the hMSC sIPN constructs three times with PBS, 100 µl of the 5 mM p-nitrophenyl phosphate (pNPP) solution was added to the constructs and incubated for 60 min at 37°C in dark, and then 100 µL of stop solution was added. The absorbance was measured using a multiplate reader, and ALP activity was normalized by cell number using the results of the proliferation assay. NPP concentration standards ranging from 0–20 nmol were used to verify linearity of the measurements over the range of the assay.

#### 5.3. Analysis of osteogenic gene expression

Relative protein expression of runt-related transcription factor 2 (RUNX2), integrin-binding sialoprotein (iBSP) and osteocalcien (OCN) by hMSCs was performed as described previously for STRO-1. Briefly, fixed MSCs were incubated with human anit-iBSP monoclonal mouse IgG antibodies (1∶50 dilution), human anti-RUNX2 monocoloal mouse IgG antibody (dilution 1∶50) or human anti-OCN monocoloal mouse IgG antibody (1∶50 dilution) overnight at 4°C. After washing three times with PBS, goat anti-mouse IgG-HRP secondary antibody (1∶100 dilution) was added to the samples, and incubated for 2 hr at room temperature. Then, hMSCs were incubated with TMB substrate for 5 minute at room temperature and absorbance values of supernatants at 450 nm were recorded using a multiplate reader. Protein expression was normalized by cell number using the results of the proliferation assay.

#### 5.4. Assessment of matrix calcification

To localize calcium within the matrix, cells were fixed with 4% paraformaldehyde for 15 minutes at room temperature and then incubated with 2 wt% Alizarin red S in DI water (pH 4.0) for 15 min at room temperature. After extensive washing with DI water, samples were imaged with a Nikon optical microscope. To quantify the calcium in the matrix, the hMSC sIPN constructs were rinsed three times with PBS, incubated with 0.5 M HCl for 24 h at 4°C, and then secreted calcium in the matrix was extracted by vortexing. The hydrogel, matrix, and cell material were collected by centrifugation, and calcium content in the supernatant was measured using a calcium detection kit (Cayman Chemical, Ann Arbor, MI) following the manufacturer's protocol. Absorbance of the samples at 570 nm was measured on a multiplate reader. Total calcium in solution was calculated from calcium concentration standards prepared in parallel with the assay and normalized by cell number using the results of the proliferation assay.

#### 5.5. Analysis of type I collagen expression

Localization of secreted type I collagen by the hMSCs was observed by fixing the cells as described previously, blocking with 3 wt% BSA in PBS and incubated with human anit-collagen type I monocoloal mouse IgG (1∶100 dilution) at 4°C overnight. After washing three times with PBS, gel disks were incubated with goat Texas Red-labeled polycolonal anti-mouse IgG secondary antibody (1∶100 dilution) for 2 hours. Finally, cell nuclei were stained with DAPI (1∶1000) for 5 min at room temperature. Confocal images were acquired using a two photon/confocal microscope. Quantification of type I collagen was performed using the hydroxyproline assay. hMSC-sIPN constructs were hydrolyzed in 2N HCl for 18 h at 110°C, and then the pH of the solution was neutralized with 2.5 N NaOH and 0.5 N HCl. Separately, chloramine-T was dissolved in a citrate buffer (pH 6)/isopropanol mixture (8∶1 v/v) at a concentration 15.7 mg/mL, and the p-DAB solution was prepared by dissolving p-DAB in isopropanol (30 mL) and perchloric acid (60%, 13 mL) at a concentration of 174 mg/mL. 100 µL of the neutralized sample digests were mixed with 50 µL of chloramine-T for 15 min at room temperature. 50 µL of p-DAB solution was then added to the solution, and the plate was incubated at 37°C for 45 minutes in dark. The absorbance of the final solution was detected at 550 nm using a multiplate reader, compared against the absorbance measured from standard solutions of type I collagen at concentrations ranging from 0 to 100 µg/mL and normalized by cell number using the results of the proliferation assay.

### 6. Statistical analysis

All quantitative measurements were performed on at least triplicate sIPNs. All values are expressed as mean ± standard deviation. One-way ANOVA with *post-hoc* Tukey tests were used to compare treatment grous in the quantitative measurements and p<0.05 was used to assess statistical significance.

## Results and Discussion

### 1. Synthesis of sIPN and hMSC sensitivity to sIPN parameters

**Figure 1:**
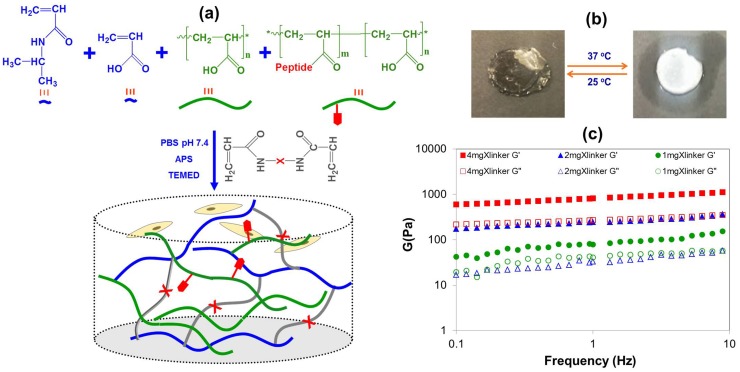
Synthesis and characterization of sIPN hydrogels. (**a**) Schematic for sIPN synthesis: a solution of NIPAAm, AAc, and QPQGLAK diacrylate in PBS containing p(AAc)-*g*-RGD undergoes free radical polymerization to form a sIPN of p(NIPAAm-co-AAc) crosslinked with an MMP-13 degradable peptide crosslinker, and interpenetrated with p(AAc)-*g*-RGD. (**b**) Evidence of phase transition behavior by sIPNs. (**c**) Rheological properties of the sIPNs were determined using various crosslinking densities at 37°C. The filled symbol represents the storage modulus (G′), and the open symbol represents the loss modulus (G"). Three repeated measurements were performed on each sample.

Thermoresponsive p(NIPAAM)-based sIPNs were synthesized as described previously to *independently* control their mechanical and biological properties (**Figure 1a**). Acrylic acid (AAc) was included in the sIPNs to control the water retention, volumetric change upon transition, and the lower critical solution temperature (LCST), and the same amount of AAc was used for all of the sIPNs in this study (**Figure 1b**). Hydrogels with different degrees of stiffness were generated by varying the amount of crosslinker added during synthesis, and the storage moduli (G′) for these hydrogels were measured to be 102, 390 and 970 Pa (**Figure 1c**). Three different concentrations of bsp-RGD(15) (i.e., 0, 105 or 210 µM) were added to these hydrogels to yield 9 material substrates with different stiffnesses and adhesion ligand concentrations.

**Figure 2:**
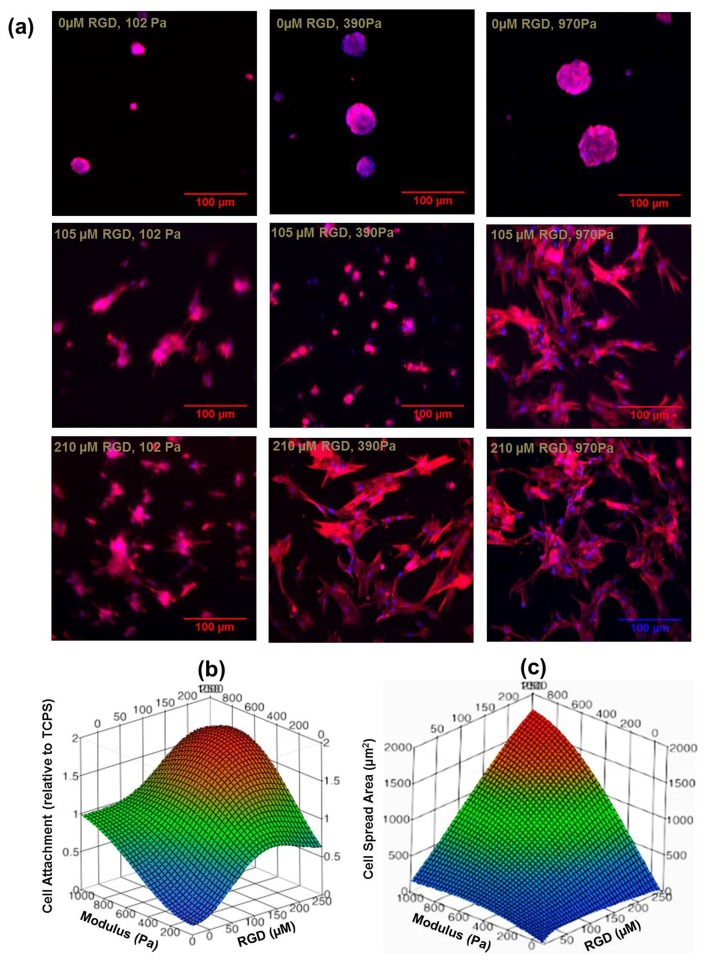
Cell adhesion and spreading on the sIPN hydrogels. (**a**) hMSCs were capable of adhering and spreading on the sIPNs containing the bsp-RGD(15) adhesive ligand, as assessed by f-actin stress fibers (TRITC-phalloidin; red) and nuclei (DAPI; blue). Scale bar  = 100 µm. RSM plots demonstrating the effect of sIPN stiffness (102–970 Pa) and bsp-RGD(15) adhesive ligand density (0–210 µM) on: (**b**) hMSC attachment after 24 hours relative to cells seeded on TCPS; (**c**) and area of hMSC spreading after 3 days.

All of the sIPNs substrates supported hMSC viability regardless of their material parameters (**Figure S1**), although cell morphology (i.e., spreading) was strongly dependent on the matrix parameters (**Figure 2a**). Cell attachment to the substrate within 3 days of seeding was significantly lower for sIPNs with 102 Pa stiffness and 0 µM bsp-RGD(15) relative to any other matrix parameters (**Figure S2a**), and cell attachment appeared to correlate with both the stiffness and bsp-RGD(15) concentration in all the other sIPNs (**Figure 2b**). Cells exhibited primarily a rounded morphology on the substrates with either the lowest bsp-RGD(15) concentration (0 µM) or stiffness (102 Pa). Cell spreading areas on these sIPNs was negligible (**Figure 2c**) and significantly lower than for the other sIPNs (**Figure S2b**). Importantly, cell spreading on 3 of the matrices (210 µM and 970Pa; 210 µM and 390Pa; and 105 µM and 970 Pa) was higher than cell spreading on TCPS, and the greatest cell spreading was observed on matrices with the highest bsp-RGD(15) ligand density (210 µM) and stiffness (970 Pa) (**Figure. S2b, S3**).

**Figure 3:**
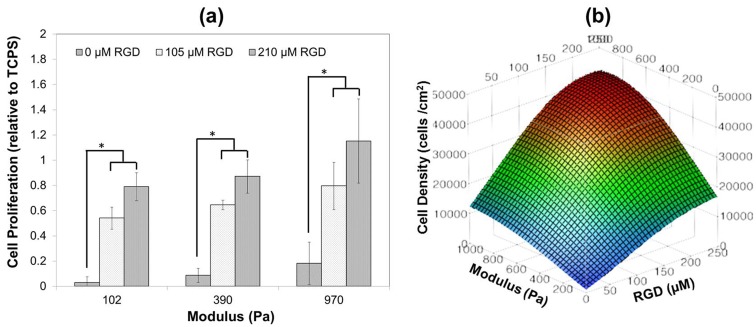
Cell proliferation on the hydrogels. (**a**) hMSC proliferation after 21 days in growth media relative to TCPS mean data (40,458 cells/cm^2^) demonstrating the effect of sIPN stiffness (102–970 Pa) and RGD adhesive ligand density (0–210 µM). (**b**) RSM plots demonstrating the effect of sIPN stiffness (102–970 Pa) and bsp-RGD(15) adhesive ligand density (0–210 µM) on hMSC proliferation after 21 days in growth media.

Proliferation onto the hydrogels for hMSCs was assessed 21 days after attachment to each of the sIPN substrates (**Figure 3a**). Minimal hMSC growth was observed for any of the sIPNs with 0 µM bsp-RGD(15), and cell proliferation was correlated with both substrate stiffness and RGD density within the range we evaluated and approached a plateau at the maximum parameters (i.e., 210 µM and 970 Pa), which was within the 95% confidence interval for cell proliferation on TCPS (4 GPa [36], (**Figure 3b**). The effects of peptide density and substrate stiffness on cell proliferation appeared to be synergistic within this range.

Upon cell adhesion to the substrate, hMSCs sense the underlying matrix and respond to the biochemical and mechanical matrix factors that modulate subsequent processes such as spreading, proliferation, and migration. Our findings are supported by previous reports demonstrating that proliferation is dependent on both cell adhesion peptide density, peptide affinity, and matrix stiffness for several cell types [22], [37], [38], [39], [40], including MC3T3-E1 preosteoblasts [5]. Our previous work has also demonstrated that proliferation of rat calvarial osteoblasts on p(NIPAAm-co-AAc) hydrogels was correlated with substrate stiffness [13]. Furthermore, hMSC function was dependent on their ability to generate sufficient cytoskeleton tension [41], [42], and osteogenic differentiation of hMSCs depended on actin cytoskeleton spreading and focal adhesion assembly [43], [44]. Therefore, further investigation of the sIPNs in this study was focused on the four substrates that were sufficient to promote hMSC spreading and cytoskeleton formation (see **Table 1**) to determine the relative effect of the matrix parameters during osteogenic induction.

**Table 1:** Matrix parameters for sIPN used in hMSC osteogenic differentiation assays.

sIPNs	Modulus (Pa)	Peptide Density (µM)
Low Modulus Low Peptide Density	390	105
Low Modulus High Peptide Density	390	210
High Modulus Low Peptide Density	970	105
High Modulus High Peptide Density	970	210

### 2. Osteogenic differentiation of hMSC on sIPNs

**Figure 4:**
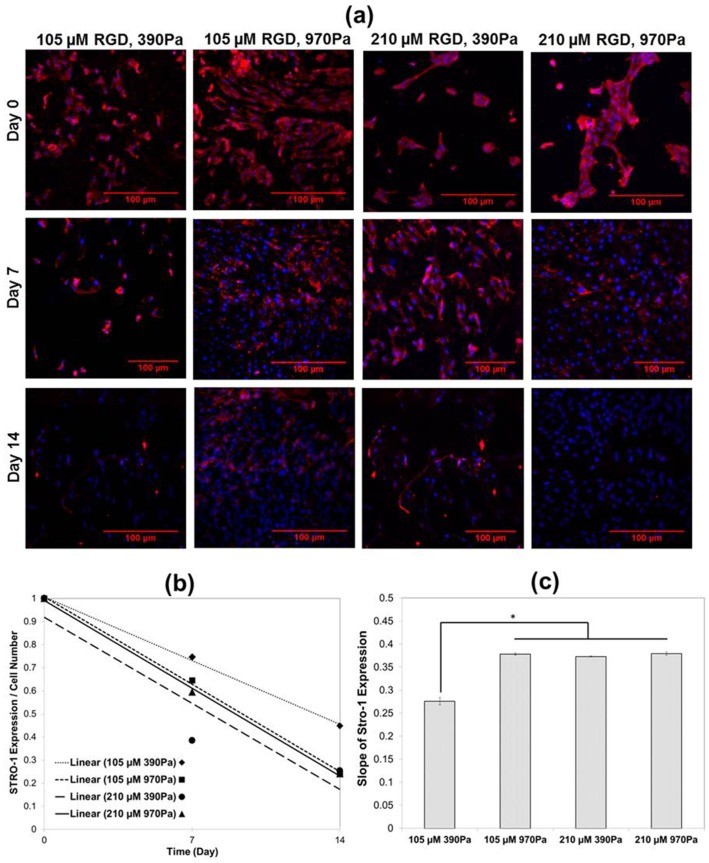
Dependence of cell differentiation on stiffness and adhesion peptide density. (**a**) Representative STRO-1 staining of hMSCs after culture in osteogenic induction media for 0, 7 and 14 days on various sIPNs, as assessed by STRO-1 positive cells (red) and nuclei (DAPI; blue). Scale bar  = 100 µm. (**b**) Relative time dependent expression of STRO-1 on various sIPNs. (**c**) Rate of decrease (i.e., slope of correlation in panel b) of STRO-1 protein expression. *p<0.05, ANOVA and Tukey *post-hoc* tests with n = 3 sIPNs.

Concurrent with hMSC differentiation, cell-surface expression of the stemness marker STRO-1 was down regulated and served as an indicator of decreased hMSC cell lineage commitment (**Figure 4a**) [45], [46]. Maximal expression of STRO-1 was measured on the first day of osteogenic induction, and the expression of STRO-1 was steadily downregulated during the first 14 days of culture under osteogenic differentiation conditions for cells on all of the sIPNs substrates (**Figure 4b**). The rate (i.e., slope) of decreasing STRO-1 expression was similar for hMSCs on any of the sIPN substrates containing either the higher peptide density or higher substrate stiffness, but this rate was significantly different for hMSCs on the sIPN with the lowest peptide density and stiffness (**Figure 4c**). By comparison, STRO-1 expression on TCPS was visible even after the 21 days of culture (**Figure S3**).

**Figure 5:**
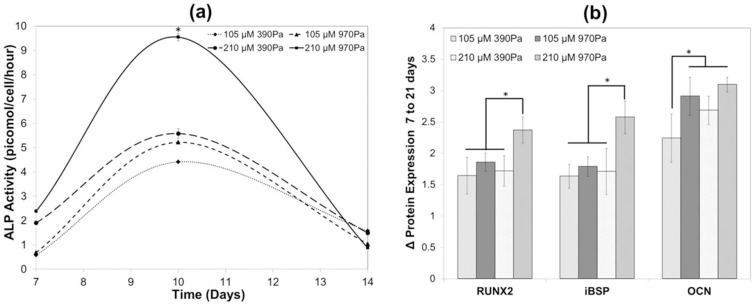
Relative osteogenesis on the various sIPNs. (**a**) Quantitative analysis of ALP activity, and (**b**) RUNX2, iBSP and OCN protein expression on various sIPNs. *p<0.05, ANOVA and Tukey *post-hoc* tests with n = 3 sIPNs.

Thereafter, progression of osteogenic differentiation was assessed by quantification of ALP activity, protein expression analysis of the osteogenic transcriptional regulator RUNX2, and the secreted non-collagenous ostegenic matrix proteins iBSP and OCN [47], [48], [49]. Our results are consistent with previous studies showing maximal ALP expression between days 7 and 10 [50], [51]. sIPNs with 210 µM peptide densities and 970 Pa matrix stiffness yielded the greatest ALP expression, but sIPNs containing either of the higher matrix parameters enhanced ALP expression relative to those containing both the low (105 µM) bsp-RGD(15) peptide density and the low (390 Pa) matrix stiffness (**Figure 5a**). Fourteen days after the start of osteogenic differentiation, the highest expression of RUNX2 and iBSP was observed on the matrix containing the highest (210 µM) bsp-RGD(15) peptide density and the highest (970 Pa) matrix stiffness, and a similar, lower expression level was observed on the other sIPNs (**Figure 5b**). By contrast, the expression of OCN on all of the sIPNs was approximately the same for all sIPNs after 14 days, except for the matrix containing the lowest bsp-RGD(15) concentration (105 µM) and matrix stiffness (390 Pa). We attribute this observation to the proteins iBSP and RUNX2 being upregulated during early bone formation, whereas OCN is expressed at the later time point of osteogenesis [52].

**Figure 6:**
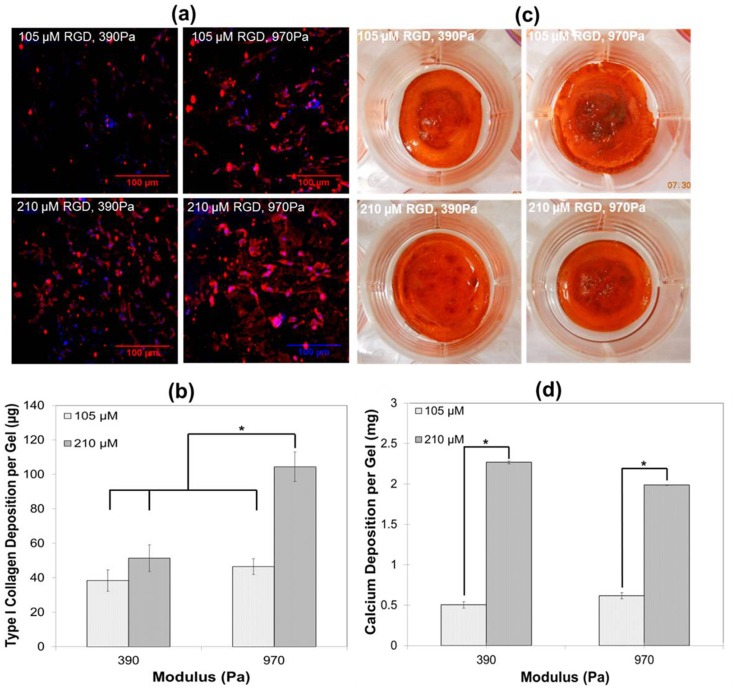
Quantitative analysis of secreted type I collagen and calcium on sIPNs. Type I collagen protein expression was determined (**a**) qualitatively by immunostaining and (**b**) quantitatively by the hydroxyproline assay on various sIPNs. Osteogenesis was characterized by calcium deposition on the matrix by (**c**) Alizarin red S staining and (**d**) quantified using a calcium detection kit on various sIPNs. *p<0.05, ANOVA and Tukey *post-hoc* tests with n = 3 sIPNs.

As a final indicator of osteogenic differentiation, we assessed the ability the sIPNs substrates to promote the formation of a collagen–proteoglycan rich matrix that was able to mineralize *in vitro*. Immunostaining of type I collagen was positive on all four sIPNs, indicating that the cells had deposited a collagen rich matrix (**Figure 6a**), but collagen expression was maximized by the sIPN with the highest (210 µM) bsp-RGD(15) concentration and matrix stiffness (970 Pa; **Figure 6b**). Alizarin red staining confirmed the presence of calcium binding to the matrix 21 days after osteogenic induction (**Figure 6c**). Both of the substrates containing the higher peptide concentration contained significantly higher calcium content relative to those with the lower bsp-RGD(15) concentration, and this result was independent of the substrate stiffness (**Figure 6d**). Minimal osteogenesis of hMSCs was observed on matrices without RGD peptide and stiff TCPS substrates as confirmed by ALP activity, protein expression, calcium and type I collagen production (**Figure S3c, S3d, S4, and S5**).

Our results indicate that osteogenic differentiation of hMSCs can occur with similar efficiency on compliant substrates compared to stiff substrates; however, under specific media conditions differentiation can be controlled by either the substrate stiffness or adhesive ligand density. Our findings are also consistent with ample literature reports indicating that cell shape, for which actomyosin contractility is a prerequisite, plays a key role in regulating the functional activities of stem cells [53], including proliferation and differentiation [52], [54], [55], [56], [57], [58]. Similarly, cell spreading on bsp-RGD(15)-presenting substrates, ALP activity, OCN, iBSP gene expression have been correlated with increasing substrate stiffness (>13.7 kPa) using MC3T3-E1 preosteoblasts [59]. In our study, we encouraged cell spreading on the substrates by increasing either the matrix bsp-RGD(15) concentration or stiffness, and consistent with other reports, both methods supported higher levels of osteogenic differentiation [52]. These findings also support our previous observations demonstrating significant bone formation in an *in vivo* marrow ablation study using sIPNs with 500 µM bsp-RGD(15) adhesion ligand and 500 Pa modulus [13]. Taken together, our findings suggest that for hMSCs in compliant, low-modulus tissues or matrices, such as those relevant to intramembranous bone formation and injectable biomaterials, the adhesive ligand affinity and presentation are critical parameters to compensate for the absence of a rigid substrate by ensuring sufficient cell spreading for osteogenic function.

## Conclusions

To develop *in situ* regenerative therapies for bone tissue engineering, there is an unmet need to study osteogenesis on soft injectable substrates. Here, we have shown that thermoresponsive soft injectable sIPNs can support survival, proliferation and osteogenesis of hMSCs, and that the matrix stiffness and adhesive ligand density can be used to control cell-substrate attachment, cell proliferation and osteogenic differentiation. Further, we have verified osteogenic differentiation within these compliant matrices, as confirmed by the upregulation of osteogenic proteins RUNX2, iBSP, and OCN, and mineralized collagenous matrix formation. Finally, we have explored the interaction between matrix compliance and adhesive ligand density as a strategy to compensate for the lack of a rigid substrate by encouraging sufficient cell spreading, leading to robust osteogenic differentiation. The findings of this study are important for understanding the basic biology of osteogenesis within compliant tissue matrices, as occurs during intramembranous bone formation, and are also useful for the development of *in situ* forming biomaterials for bone tissue engineering.

## Supporting Information

Figure S1 **Cell viability on sIPNs.** Representative live/dead staining of hMSCs cultured on various sIPNs was high after one day of culture, as assessed by double staining with calcein (green, live cells) and propidium iodide (red, dead cells).
(TIF)

Figure S2 **Cell adhesion and morphology.** Plots demonstrating the effect of sIPN stiffness (102–970 Pa) and RGD adhesive ligand density (0–210 µM) relative to TCPS on (**a**) hMSC attachment after 24 hours (TCPS mean  = 7,359 cells/cm^2^), and (**b**) area of hMSC spreading after 3 days (TCPS mean  = 651 µm^2^).
(TIF)

Figure S3 **Human MSC culture on TCPS.** (**a**) Immunostaining of STRO-1 of hMSCs on TCPS at day 0, 7, and 14. (**b**) F-actin stress fibers (TRITC-phalloidin; red) and nuclei (DAPI; blue) at day 3. (**c**) Immunostaining of secreted type I collagen (red) on TCPS. (**d**) Alizarin red S staining to determine the calcium production. Scale bar  = 500 µm.
(TIF)

Figure S4 **Relative osteogenesis on various sIPNs.** (**a**) Quantitative analysis of ALP activity, and (**b**) RUNX2, iBSP and OCN protein expression on various sIPNs containing 0 µM bsp-RGD(15).
(TIF)

Figure S5 **Analysis of secreted type I collagen and calcium on sIPNs**. Type I collagen protein expression was determined (**a**) qualitatively by immunostaining and (**b**) quantitatively by the hydroxyproline assay on various sIPNs containing 0 µM bsp-RGD(15). Osteogenesis was characterized by calcium deposition on the matrix (**c**) qualitatively by Alizarin Red S staining and (**d**) quantitatively using a calcium detection kit on various sIPNs containing 0 µM bsp-RGD(15).
(TIF)
