# Supplementary figures and images for: Controlling Osteogenic Stem Cell Differentiation via Soft Bioinspired Hydrogels
Source: PLoS One. 2014 Jun 17;9(6):e98640. doi: 10.1371/journal.pone.0098640 (PMC4060996; doi:10.1371/journal.pone.0098640)

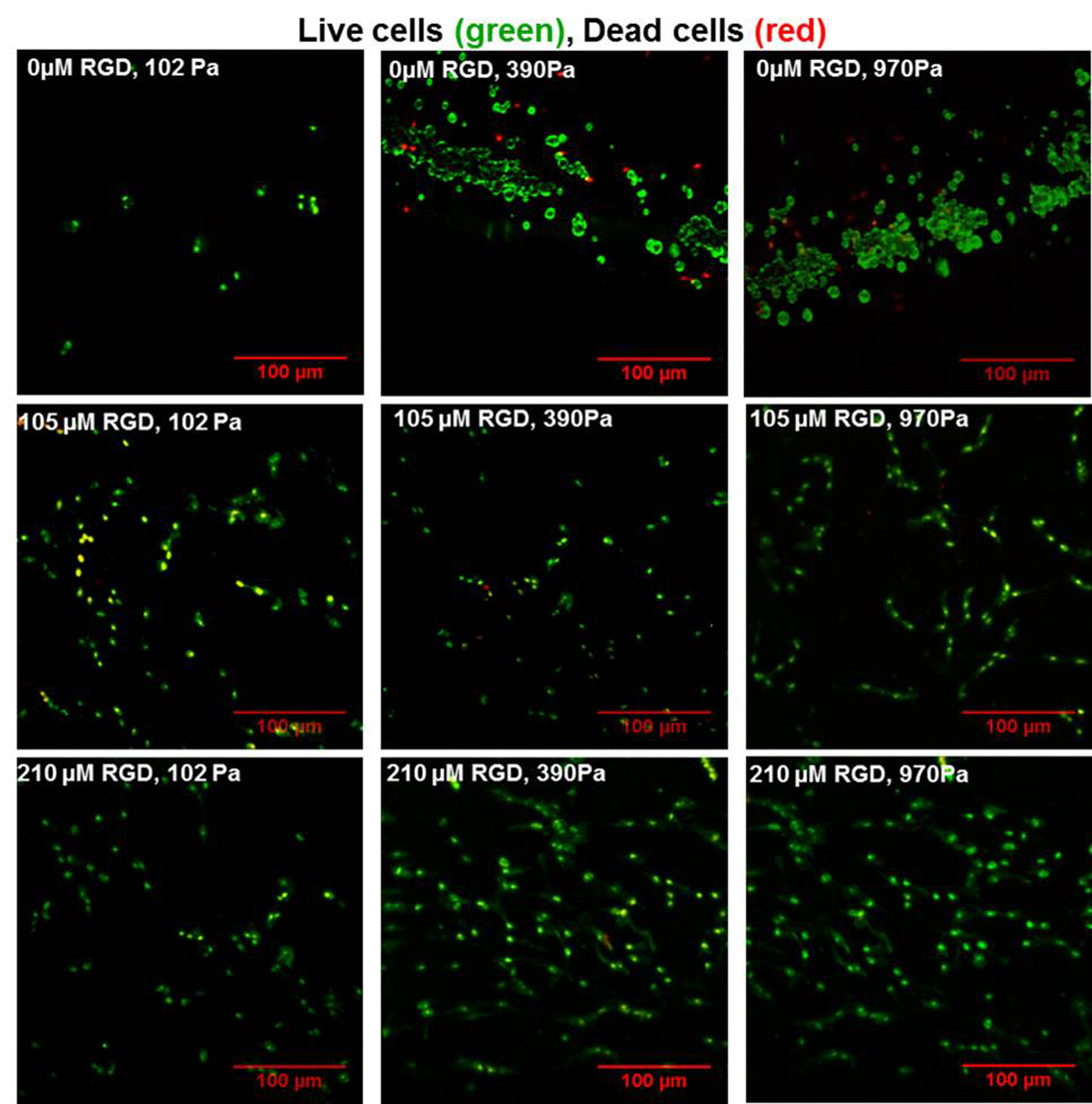

Supplement: Figure S1 — Cell viability on sIPNs. Representative live/dead staining of hMSCs cultured on various sIPNs was high after one day of culture, as assessed by double staining with calcein (green, live cells) and propidium iodide (red, dead cells). (TIF) [file pone.0098640.s001.tif]

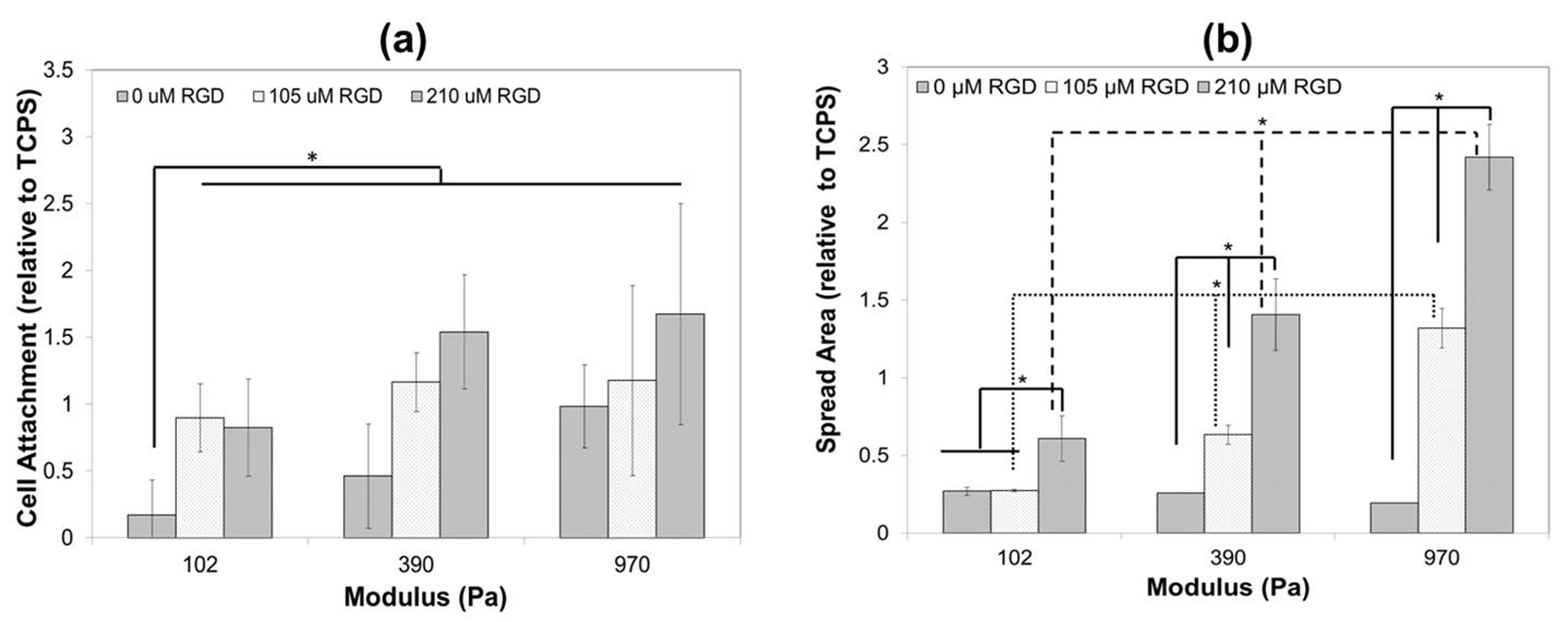

Supplement: Figure S2 — Cell adhesion and morphology. Plots demonstrating the effect of sIPN stiffness (102–970 Pa) and RGD adhesive ligand density (0–210 µM) relative to TCPS on (a) hMSC attachment after 24 hours (TCPS mean = 7,359 cells/cm2), and (b) area of hMSC spreading after 3 days (TCPS mean = 651 µm2). (TIF) [file pone.0098640.s002.tif]

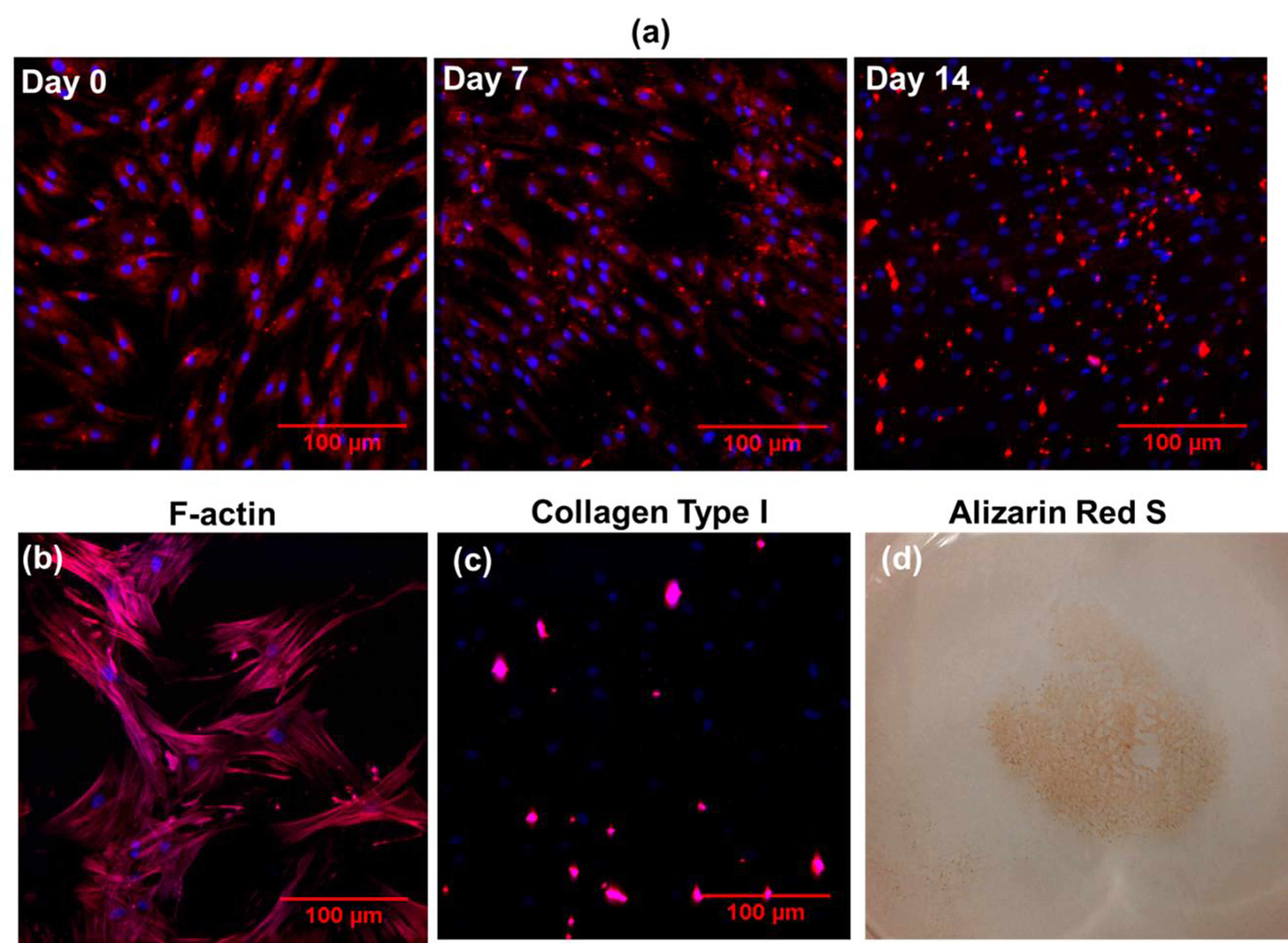

Supplement: Figure S3 — Human MSC culture on TCPS. (a) Immunostaining of STRO-1 of hMSCs on TCPS at day 0, 7, and 14. (b) F-actin stress fibers (TRITC-phalloidin; red) and nuclei (DAPI; blue) at day 3. (c) Immunostaining of secreted type I collagen (red) on TCPS. (d) Alizarin red S staining to determine the calcium production. Scale bar = 500 µm. (TIF) [file pone.0098640.s003.tif]

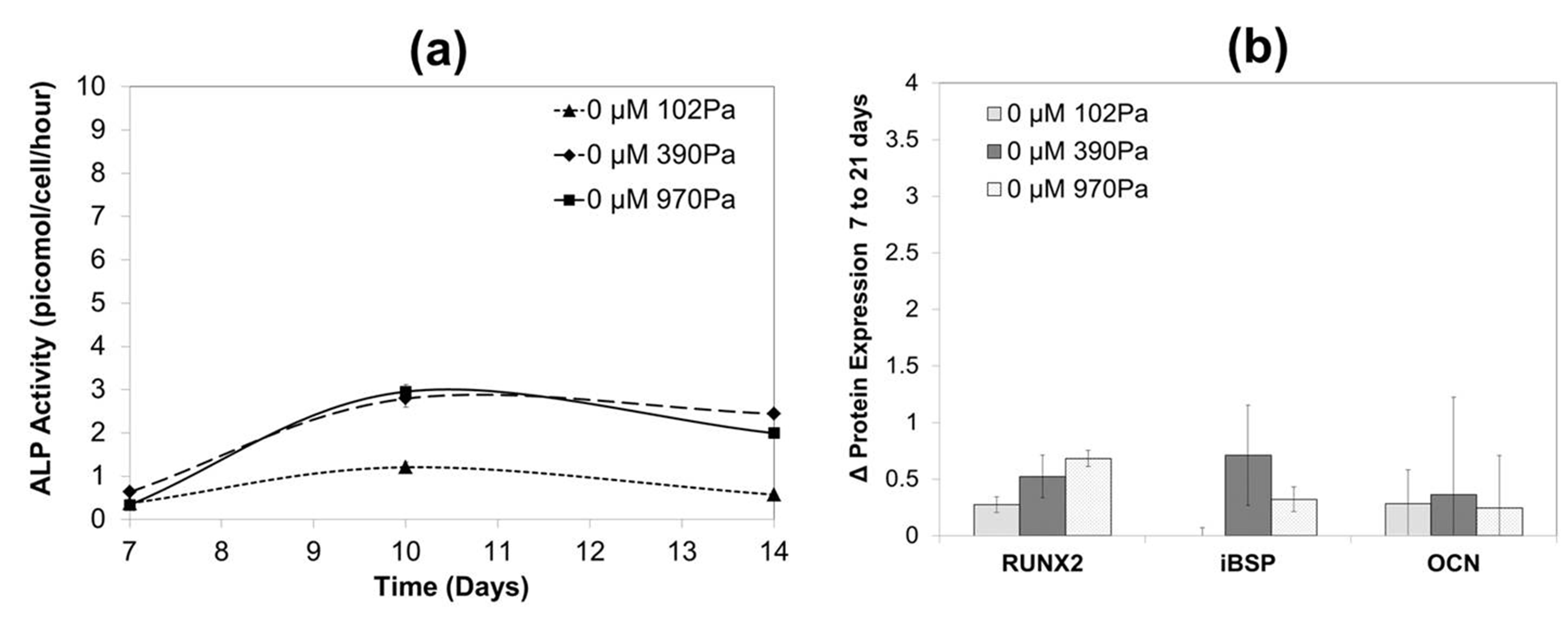

Supplement: Figure S4 — Relative osteogenesis on various sIPNs. (a) Quantitative analysis of ALP activity, and (b) RUNX2, iBSP and OCN protein expression on various sIPNs containing 0 µM bsp-RGD(15). (TIF) [file pone.0098640.s004.tif]

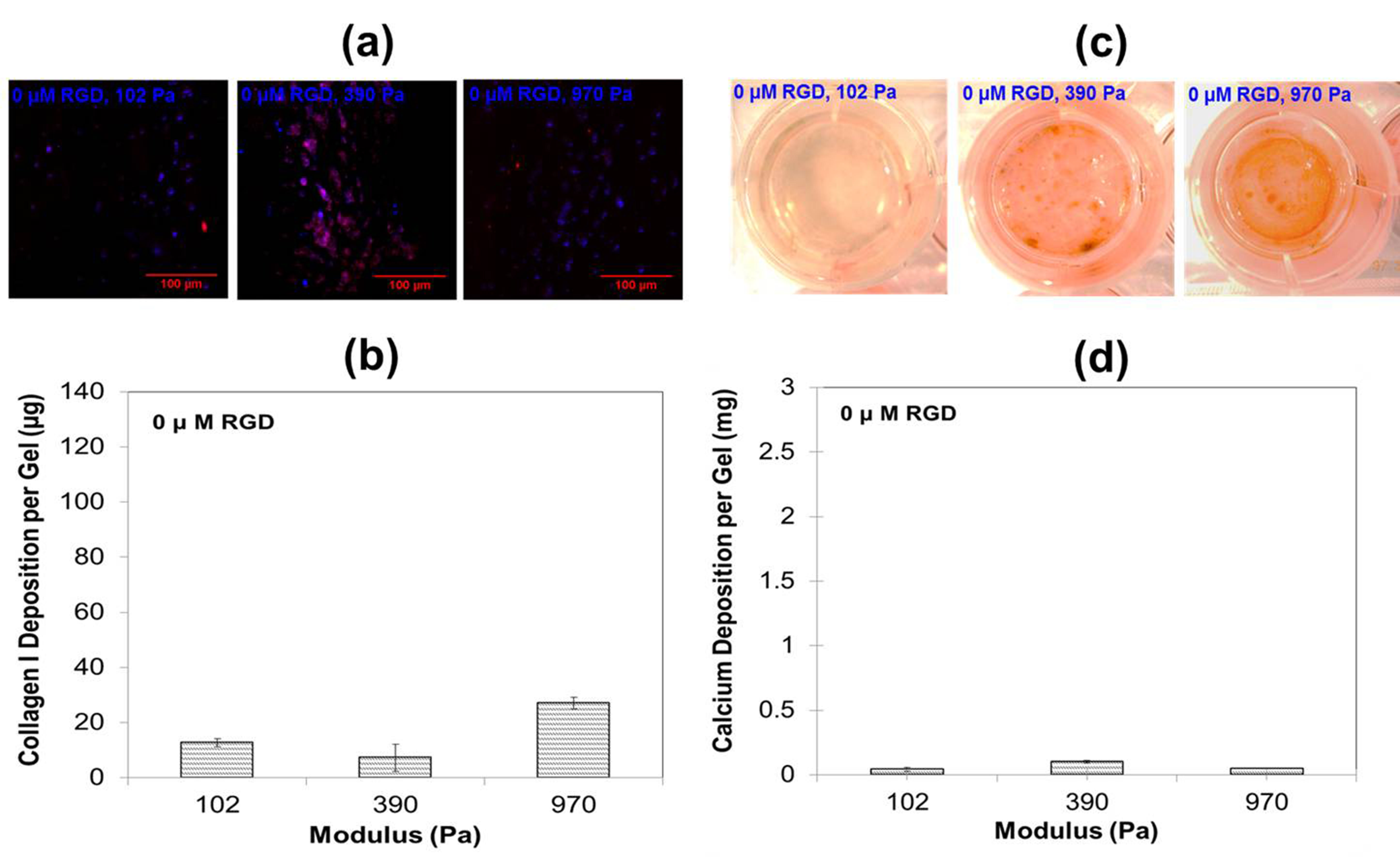

Supplement: Figure S5 — Analysis of secreted type I collagen and calcium on sIPNs. Type I collagen protein expression was determined (a) qualitatively by immunostaining and (b) quantitatively by the hydroxyproline assay on various sIPNs containing 0 µM bsp-RGD(15). Osteogenesis was characterized by calcium deposition on the matrix (c) qualitatively by Alizarin Red S staining and (d) quantitatively using a calcium detection kit on various sIPNs containing 0 µM bsp-RGD(15). (TIF) [file pone.0098640.s005.tif]
